# The Tick Microbiota Dysbiosis Promote Tick-Borne Pathogen Transstadial Transmission in a *Babesia microti*–Infected Mouse Model

**DOI:** 10.3389/fcimb.2021.713466

**Published:** 2021-08-03

**Authors:** Nana Wei, Jie Cao, Houshuang Zhang, Yongzhi Zhou, Jinlin Zhou

**Affiliations:** Key Laboratory of Animal Parasitology of Ministry of Agriculture, Shanghai Veterinary Research Institute, Chinese Academy of Agricultural Sciences, Shanghai, China

**Keywords:** *Haemaphysalis longicornis*, microbiota dysbiosis, antibiotic usage, *Babesia microti*, peritrophic matrix

## Abstract

Ticks are obligate hematophagous ectoparasites. They are important vectors for many pathogens, of both medical and veterinary importance. Antibiotic residues in animal food are known, but very little is known about the effects of antibiotic residues in animals on the microbiome diversity of ticks and tick-borne pathogen transmission. We used a *Haemaphysalis longicornis*–infested mouse model to evaluate the effect of antibiotic usage on tick microbiome. Nymphal ticks were fed on an antibiotic cocktail-treated or water control mice. Adult ticks molted from nymphs fed on the antibiotic cocktail-treated mouse had a dysbiosed microbiota. Nymphal ticks were also fed on a *B. microti*–infected mice that had been treated with antibiotic cocktail or water. We found that the *B. microti* infection in adult ticks with a dysbiosed microbiota (44.7%) was increased compared with the control adult ticks (24.2%) by using qPCR targeting 18S rRNA gene. This may increase the risk of tick-borne pathogens (TBPs) transmission from adult ticks to a vertebrate host. These results show that an antibiotic-treated mouse can induce tick microbiota dysbiosis. Antibiotic treatment of *B. microti*-infected mouse poses the possibility of increasing transstadial transmission of *B. microti* from the nymph to the adult *H. longicornis*. These findings suggest that *B. microti* transmission may be exacerbated in high antibiotic usage areas.

## Introduction

Ticks are obligate hematophagous ectoparasites of vertebrates, which feed on a wide range of hosts, including mammals, birds, and reptiles. Ticks are major vectors of many pathogens, especially zoonotic pathogens. Ticks are important vectors of human infectious diseases worldwide and are second only to mosquitoes in terms of public health importance. Ticks are responsible for the transmission of infectious agents, such as bacteria (*Coxiella*, *Rickettsia*, *Borrelia*, and *Anaplasma*), viruses (severe fever with thrombocytopenia syndrome virus, tick-borne encephalitis virus, and Alongshan virus), and parasites (*Babesia*, and *Theileria*).

The four development stages of ticks are eggs, larvae, nymphs, and adults. All mobile life stages bite humans and animals. Nymphs and adult females are most commonly encountered. Nymphs and adults are also the most important stages for the transmission of tick-borne pathogens (TBPs). Nymphs are one of the most dangerous tick stage, because they are small and difficult to detect. Few TBPs are transmitted transovarially, and most TBDs are transmitted by nymphs or adult ticks (Bonnet et al., 2017). The ixodid ticks typically feed only once during each developmental stage, after which they detach from the host. Unfed adult ticks do not acquire pathogens from nymphs. Pathogens are transstadially transmitted from nymphs to adults. Furthermore, adult ticks effectively transmit pathogens to vertebrate hosts during blood feeding. The study of transstadial transmission of TBPs from nymphs to adults is significant for tick and TBD control.

Microbial communities within vectors are important in pathogen colonization. They subsist in vectors and can influence pathogen transmission from vectors to hosts (Gall et al., 2016; Abraham et al., 2017; Rodgers et al., 2017). The bacterial microbiome of *Dermacentor andersoni* ticks influences pathogen susceptibility (Gall et al., 2016). The blood meal source significantly influences tick microbiomes and may impact pathogen transmission (Swei and Kwan, 2017). *Ixodes scapularis* ticks do not harbor a stable midgut microbiome (Ross et al., 2018). In *Anopheles* mosquitoes, malaria parasite infection increased when gut microbiota colonization was impaired by feeding on an antibiotic-treated sugar solution (Dong et al., 2009; Meister et al., 2009). Antimicrobial agents and antibiotics have been widely used on animal farms for treatment of animal diseases. Antibiotics are also commonly used to treat skin lesions and TBD associated with tick bites, such as Lyme disease on humans. Tick populations and TBD are increasing, and newly emerging TBPs are being reported. The antibiotics present in the blood meal of ticks could potentially interfere with the tick microbiome and result in tick gut dysbiosis. This may enhance tick susceptibility to pathogens and promote TBP transmission.

To test this possibility, we used an *H. longicornis* infestation model and a *B. microti* infection model in mice. *H. longicornis* is an important tick species that maintains and transmits a variety of zoonotic pathogen agents, including *B. microti*, severe fever with thrombocytopenia virus (SFTSV), *Rickettsia raoultii*, *Ehrlichia chaffeensis*, and *Borrrlia garinii*. *H. longicornis* is a new and emerging threat to public health in US states (Tanne, 2018). Babesiosis is a worldwide, malaria-like tick-borne zoonotic infectious disease that can lead to livestock losses and health issues for humans. The genus *Babesia*, including obligate intraerythrocytic protozoans, includes the causative agents of babesiosis. *B. microti* is the most prevalent species associated with human babesiosis in the USA and in study regions of the People’s Republic of China (Vannier and Krause, 2012; Ord and Lobo, 2015).

We previously demonstrated that tick nymphs fed on *B. microti*–infected mice could molt into infected adult ticks (Wu et al., 2017). *B. microti* is typically transmitted to the host during the blood meal. This feeding strategy provides an ideal pathogen model to study the tick microbiome and its effects on pathogen transmission by the tick vector. In our antibiotic model for mice treatment, we used a cocktail of ampicillin, metronidazole, neomycin trisulfate, and vancomycin, which are all important antimicrobials in human and veterinary medicine (World Health Organization, 2017).

## Materials and Methods

### Ethics Statement

All animal experimental protocols in this study were approved by the Institutional Animal Care and Use Committee of Shanghai Veterinary Research Institute (IACUC approval number: SHVRI-SZ-20200608-01). After approval, the experiment was carried out in compliance with the Guidelines on the Humane Treatment of Laboratory Animals.

### Mice, Ticks, and *B. microti*

Six- to 8-week-old female BALB/c mice (18-20 g/mouse) were purchased from SPF (Beijing) Biotechnology Co., LTD (Beijing, China). The mice were housed under specific pathogen free conditions at the Animal Center of the Shanghai Veterinary Research Institute for 3 to 5 days for acclimation under standard 12-h light cycle with free access to food and water. *H. longicornis* ticks (parthenogenetic strain) were obtained from a tick colony maintained in the laboratory at 25°C with 90% relative humidity in a dark incubator. *H. longicornis* ticks used in this study were fed on BALB/c mice to keep the life cycle. The *B. microti* strain was obtained from the American Type Culture Collection (ATCC PRA-99TM; Manassas, VA, USA) and maintained in our laboratory by serial passage in BALB/c mice (Zhang et al., 2016).

### Antibiotic Administration in Mice

After the acclimation, the mice were treated with the antibiotic cocktail by intraperitoneal injection and drinking water according to a previous study with some modifications (Rodrigues et al., 2017). The control mice received only filtered water, following the same regimen. The antibiotic cocktail (1.5 g/L ampicillin, 1.5 g/L metronidazole, 1.5 g/L neomycin trisulfate, and 1 g/L vancomycin) was administered in drinking water and 100 µL of antibiotic cocktail was intraperitoneally injected at the concentration described above once each day. After about 2 weeks, nymphs were placed to feed on the mice, and the mice continued to be treated with the same antibiotic or water control administration described above until the ticks detached. Each group consisted of five mice per experiment.

### Collection of *H. longicornis* Adults

Each mouse was collared with a plastic tube with central drilling to prevent grooming and then ticks were allowed to feed to repletion on infected mice. About 35 nymphs fed to repletion on each mouse that had been treated with the antibiotic cocktail or control water. The engorged nymphs that detached from mice on the same day were collected. To prevent environmental microbial contamination, the collected ticks were surfaced sterilized with 70% ethanol twice, followed by three washes with antibiotic solution (200 µg/mL streptomycin, 200 U/mL penicillin, and 0.5 µg/mL amphotericin B), and three washes with sterilized water in a Laminar Flow Biosafety Cabinet according to the previous study with some modifications (Narasimhan et al., 2014; Swei and Kwan, 2017). They were then wicked free of excess fluid on sterile Whatman paper, placed in sterile tubes, and maintained at 25°C with 90% relative humidity in a dark sterile incubator. After two weeks, the first molted ticks were observed. The adult ticks that molted from nymphs feeding on the antibiotic or control water treated mice were designated AT adults and CT adults, respectively, in this study.

### Tick Samples Preparation and DNA Extraction

The newly molted adult ticks were collected and surface sterilized as described above to remove any surface microbial contamination. Adult ticks were dissected with sterile blades, and the guts were pooled (8 ticks) and homogenized. The supernatant was collected after centrifugation at 12,000 g for 5 min. The supernatant was used for DNA extraction using a genomic DNA Isolation Mini Kit according to manufacturer instructions (Vazyme Biotech Co., Ltd., China). DNA concentrations and purity were measured *via* a NanoDrop ND-1000 spectrophotometer (Thermo Fisher Scientific, Waltham, MA, USA). The extracted DNA was divided into two parts and stored at −80°C until further use.

### 16S rRNA Gene Profile

Sequencing of the 16S rRNA genes was performed using the Illumina MiSeq platform. Universal 16S primers (341F/806R) with a sample-specific 12 bp barcode were used to amplify the hypervariable V3-V4 region of bacterial 16S rRNA genes. PCR was performed using thermal cycler Model C1000 (Bio-Rad, Richmond, CA, USA). Microbiome bioinformatics were performed with QIIME2 2019.4 with slight modification (Bolyen et al., 2019) according to the official tutorials (https://view.qiime2.org/). Briefly, raw sequences were quality filtered, denoised, merged, and chimera filtered using the DADA2 plugin with DADA2 pipeline (Callahan et al., 2016). On average, we obtained 107445 clean reads/sample. Taxa abundances at the ASV levels were statistically compared among groups by MetagenomeSeq, and visualized as Manhattan plots (Zgadzaj et al., 2016). LEfSe (Linear discriminant analysis effect size) was performed to detect differentially abundant taxa across groups using the default parameters (Segata et al., 2011).

### Acquisition of *B. microti* by *H. longicornis* Nymphs

Blood containing *B. microti*-infected erythrocytes was collected from the mice infected with *B. microti*. Thin blood smears with Giemsa staining were used to examine the *Babesia* parasites in infected red blood cells (RBCs). About 2 weeks after the antibiotic cocktail or control water treatment, each mouse was inoculated intraperitoneally with 100 µL of *B. microti* positive blood. The parasitemia density of each mouse was monitored daily, and 35 nymph ticks were applied to feed on each mouse with *B. microti* parasitemia of 20%. The engorged nymphs were collected and surfaced sterilized as described above. The engorged nymphs from each treated group were maintained in two different dark sterile incubators until they molted into adult ticks. Fourteen days later, the first molted tick was observed.

### Detection of *B. microti* Infection in Adult Ticks

DNA was extracted from each adult tick using the method described above. Negative controls (blank samples) were used during DNA extraction. The *B. microti* infection in adult ticks was tested by qPCR targeting 18S rRNA gene to detect *B. microti* according to the previously reported method (Wu et al., 2017). Briefly, qPCR was performed to quantify *B. microti* using absolute quantification under conditions 2 min at 37°C, 30 s at 95°C, 40 cycles of 95°C for 10 s, and 60°C for 30s. A *B. microti*–positive control constructed by cloning the 429-bp PCR amplicon into a pMD18-T plasmid was used to run standard curve. All qPCR amplifications were performed in triplicate on a QuantStudio 5 Real-Time PCR machine (Applied Biosystems, Foster City, CA, USA). The 20-μl PCR mixture contained 10 μl of 2 × Probe Master Mix (QN114, Vazyme Biotech Co., Ltd., China), 1 μl of each primer, and 0.5 μl of probe at 10 μM, 3.5 μl of DNA sample, and 4 μl of water. Forward and reverse primers and probe sequences are as follows: Bm18Sf-AACAGGCATTCGCCTTGAAT, Bm18Sr-CCAACT GCTCCTATTAACCATTACTCT, and Bm18Sp-6FAM-CTA CAGCATGGAATAATGA-MGBNFQ, respectively.

### Sectioning and Staining of Adult Ticks

Adult ticks were fed for 48 hours and then dissected in 2.5% glutaraldehyde with sterile blades. The midguts were rinsed and placed in 2.5% glutaraldehyde for one hour at room temperature and overnight at 4°C. Then they were washed in graded alcohol, followed by three washes in xylene, paraffin embedded, and sectioned at about 5-μm thickness. Sections were stained with Periodic acid Schiff base (PAS) and visualized under a light microscope. The thickness of PAS positive PM-like layer was calculated in three different regions in each microscopic filed, and the arithmetic average was determined. At least three fields/section were calculated, and five to eight individual sections were examined in each treated group.

### RNAseq Library Preparation and Sequencing

Ticks were dissected and midguts were pooled (eight adult ticks) and homogenized in TRIzol (Invitrogen, USA) according to the method described above. Total RNA for RNA-seq was extracted according to the manufacturer’s instructions. Library preparation was performed using a VAHTS mRNA-seq V3 Library Prep Kit (Illumina, San Diego, CA, USA). PolyA mRNA was enriched from 1 μg of total RNA using magnetic beads with Oligo (dT). Purified mRNA was disrupted into 200 to 300 bp short fragments and primed with random hexamers for cDNA synthesis. cDNA was then subjected to end-repair and dA base addition and then connected with sequencing adapters. Suitable fragments were purified using VAHTS DNA Clean Beads (Illumina) and were selected as templates for PCR amplification. Libraries were validated with an Agilent 2100 Bioanalyzer (Agilent, Santa Clara, CA, USA) to check size distribution. Samples were qualified and pooled by qRT-PCR. Sequencing was done on Illumina novaqseq pe150.

Low-quality reads were filtered and clean reads were mapped to *H. longicornis* genome using HISAT2 software (http://ccb.jhu.edu/software/hisat2/index.shtml). Differential expression analysis was performed with the DESeq package. Genes with adjusted |log2Fold Change| > 1 and *P* value<0.05 were considered significantly differentially expressed. KEGG and GO analyses for differentially expressed genes were performed using topGO and clusterProfiler.

### Statistical Analysis

The statistical differences between experimental and control groups were determined using GraphPad Prism 8.0 software (GraphPad Software, San Jose, CA, USA). The significant differences between experiment and control groups were analyzed using Student’s *t*-test or one-way ANOVA with Dunnett’s multiple comparison post-test. A *P* value < 0.05 was considered significant.

## Results

### Antibiotic Usage on Mice Changed the Microbiota of the Newly Molted *H. longicornis* Adults

**Figure 1:**
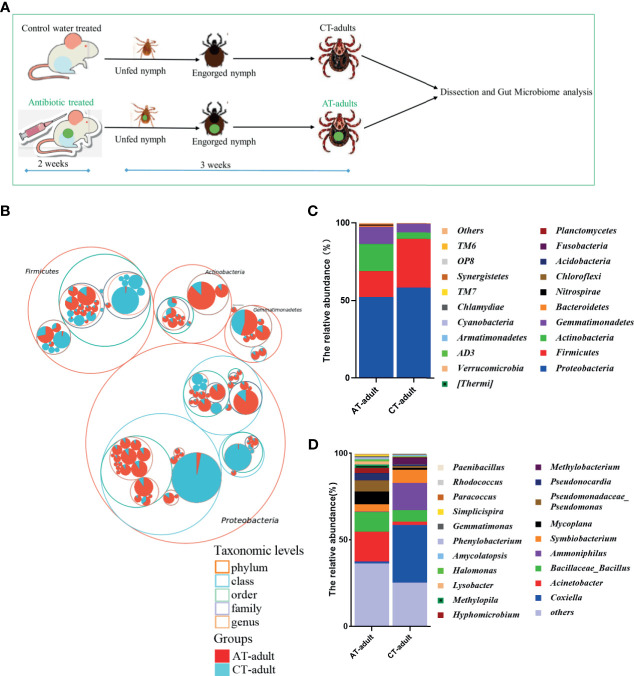
Antibiotics use on mice affects the gut microbiota community of *H. longicornis*. **(A)** The experimental workflow for microbiome analysis. Nymphs were fed on an antibiotic cocktail- treated or water control mice to generate AT adults and CT adults, respectively. The 2-week scale represents the time it takes to build an antibiotic-treated mouse model. The 3-week scale represents the time it required to obtain the newly molted adult ticks from the unfed nymphs in this study. The green dot represents perform antibiotic treatment. **(B)** Taxonomic differences are based on 16S rRNA sequencing. Taxonomic composition visualized by circular packing. The largest circles represent phylum level, and the inner circles represent class, family, and genus. The circle sizes represent the mean relative abundance of the taxa. The taxa were colored by sample groups (red for AT adults and blue for CT adults), whereas the area of the group corresponded to the mean relative abundance of the taxa in each group. **(C, D)** Total gut bacterial relative abundance at the taxonomic rank of phylum **(C)** and genus **(D)** of CT adults and AT adults.

The host blood meal source can significantly influence tick microbiomes (Swei and Kwan, 2017). Antimicrobial agents and antibiotics are widely used on animal farms to prevent and treat animal diseases. In this study, we tested the effect of antibiotics used on animals on the microbiome diversity of ticks. The tick gut is the first organ to come in contact with pathogens during blood meal uptake. It is a pivotal microbial entry point and determines pathogen colonization, survival, and infectivity (Narasimhan et al., 2014). Ticks can harbor variety of bacteria, especially in their guts. Therefore, in the present study, the gut microbiome difference between the adult ticks from different groups was determined, and the experimental workflow for microbiome analysis is shown in **Figure 1A**. Proteobacteria, Firmicutes, Actinobacteria, and Gemmatimonadetes dominated the tick gut bacterial community (**Figure 1B**), and the microbial composition of the guts of AT adults and CT adults showed differences at the phylum and genus levels (**Figures 1C, D**). The gut microbiota in AT adults was dominated by *Acinetobacter* and *Bacillaceae_Bacillus* at the genus level. However, the most abundant genera in the CT adults were *Ammoniphilus* and *Coxiella* (**Figure 1D**).

**Figure 2:**
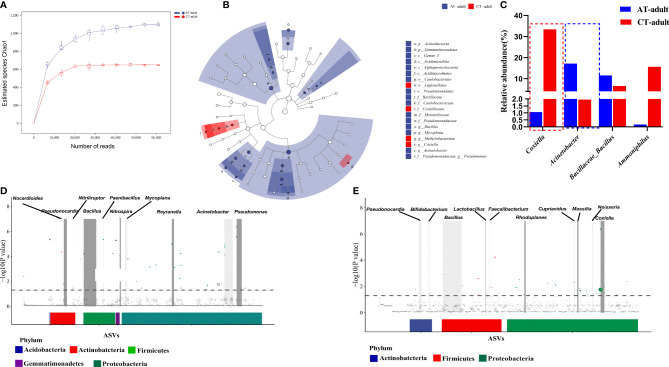
Antibiotics used on mice resulted in tick microbiota dysbiosis. **(A)** Rarefaction curve of the estimated number of genera using the Chao1 method. **(B)** Cladogram for taxonomic representation of significant differences between two groups. **(C)** Percentages of the most abundant genera in AT adults and CT adults. **(D, E)** Manhattan plots showing the abundance enrichment in the AT adults **(D)** and CT adults **(E)**. The dashed line represents the *P* = 0.05 threshold of significance. The color represents the different taxonomic affiliation of the ASVs (phylum level), and the dot size corresponds to their relative abundance in the respective samples. Gray boxes denote the different taxonomic groups (genus level).

Rarefaction curves showed that a higher bacterial diversity was observed in the guts of AT adults than in the guts of CT adults (**Figure 2A** and **Supplement Table S1**). LEfSe analysis showed that *Acinetobacter* and *Coxiella* were divided between the AT adult and CT adult groups: *Acinetobacter* in the AT adult group, and *Coxiella* in the CT adult group (**Figures 2B, C**). MetagenomeSeq analysis showed that *Nocardioides*, *Pseudonocardia*, *Bacillus*, *Paenibacillus*, *Nitrospira*, *Reyranella*, *Sphingomonas*, *Ralstonia*, *Acinetobacter*, and *Ps*eudomonas were significantly enriched in the AT adults at the genus level (**Figure 2D**), and *Pseudonocardia*, *Bifidobacterium*, *Bacillus*, *Lactobacillus*, *Faecalibacterium*, *Rhodoplanes*, *Cupriavidus*, *Massilia*, *Neisseria*, and *Coxiella* were enriched in the CT adults (**Figure 2E**). These results showed that antibiotic usage on mice changed the gut microbiota of the newly molted *H. longicornis* adults, mainly causing an increase in bacterial diversity but a decrease in bacterial abundance. The abundance of *Coxiella* and *Acinetobacter* within gut samples showed a significant difference between the two groups (**Figure 2C**), and there were different iconic species between AT adults and CT adults (**Figures 2D, E** **)**.

### Tick Microbiota Dysbiosis Influenced *B. microti* Transstadial Transmission From Nymphs to Adult Ticks

**Figure 3:**
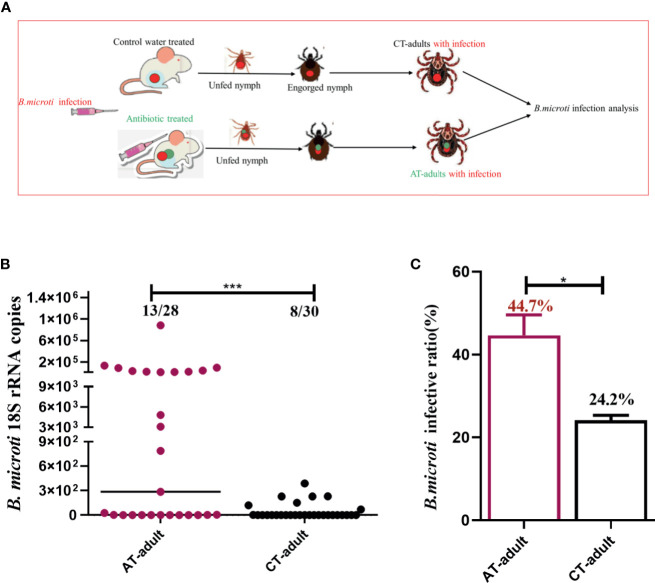
Tick microbiota dysbiosis impairs capacity to transstadially transmit *B. microti* from nymphs to adult ticks. **(A)** The experimental workflow for *B. microti* infection analysis in adult ticks. Nymphs were fed on an antibiotic cocktail-treated or water control mice with *B. microti* infection to generate AT adults with infection and CT adults with infection, respectively. **(B, C)** Assessment of the antibiotic-mediated microbiota dysbiosis regulation of tick capacity to transstadially transmit *B. microti*. * represents *p* < 0.05, *** means *p* < 0.001.

Nymphal ticks were fed on *B. microti*-positive mice treated with the antibiotic cocktail or water control, and the nymphs were collected once engorged. The infection in AT adults and CT adults was evaluated by counting the proportion of ticks carrying *B. microti* genome (prevalence) and the *B. microti* genome copies of per tick (intensity) (Melendez and Forlano, 1996). The experimental design for the *B. microti* transstadial transmission analysis is shown in **Figure 3A**. The AT adults showed a significantly increased *B. microti* prevalence and infection intensity compared to CT adults, as indicated by qPCR (**Figures 3B, C**, and **Supplement Table S2**). A 200-fold increase in *B. microti* infectivity and 1.5- to 2-fold increase in parasite prevalence occurred in AT adults (**Figures 3B, C**).

To examine the possibility that antibiotic treatment influenced *B. microti* infection and proliferation within the mice host, the parasitemia density of *B. microti* in mice at different time points after infestation with ticks was monitored. The parasitemia density of mice on the 0 and 3rd days of tick infestation was similar between the two groups (**Supplement Table S3**). These results demonstrated that the antibiotic treatment had no influence on *B. microti* infection in mice, but it did have a significant influence on the transstadial transmission from nymphs to adult ticks.

### Tick Microbiota Dysbiosis Altered Tick Gut Morphology

**Figure 4:**
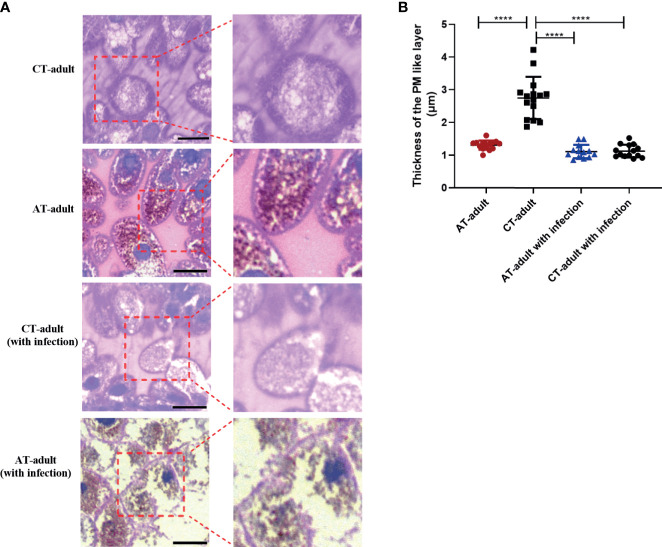
Tick microbiota dysbiosis impairs gut barrier integrity. **(A)** PAS stain and sectioned 48 h fed guts of AT adults and CT adults (under negative and positive mode). Boxed outlines within the images on the left have been magnified. The scale bar represents 50 μm. **(B)** Quantification of relative thickness of the PM-like layer of among different groups. **** means *p* < 0.0001.

The tick gut is the first organ to come in contact with pathogens. We examined the gross morphology of the tick gut. The peritrophic matrix (PM) is a glycoprotein-rich acellular structure that is located between the gut lumen and the epithelium. The tick PM is considered a barrier to parasite infection and plays an important role in host–bacteria–pathogen interactions. We determined the PM layer of adult ticks (48 h after the start of feeding on mice) using PAS staining. AT adults showed a significant decrease in the thickness of the PM compared with CT adults (**Figures 4A, B**). *B. microti* infection resulted in disruption of the PM layer in AT adults but not in CT adults (**Figure 4A**). These results indicate that tick microbiota dysbiosis induced by antibiotic usage on the mice host altered tick gut morphology. This gut morphology change may help facilitate *B. microti* transstadial transmission from nymph to adult by modulating the PM layer.

### Tick Microbiota Dysbiosis Affected the Tick Gut Transcriptional Response

**Figure 5:**
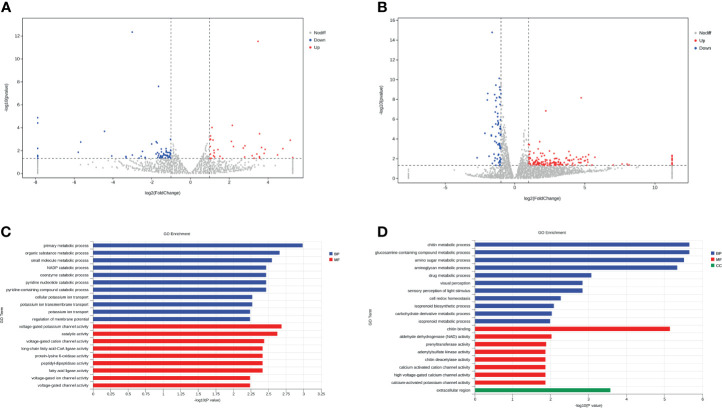
Tick microbiota dysbiosis regulates the gene expression of the tick gut. **(A, B)** Volcano plots of differentially expressed genes between the AT adults and CT adults under negative mode **(A)** and positive mode **(B)**. The up-regulated genes and down-regulated genes are shown in red and blue, respectively. **(C, D)** GO term assignments for DEGs between AT adults and CT adults under negative mode **(C)** and positive mode **(D)**. The abscissa indicates the number of unigenes in the secondary GO classification, and the ordinate indicates the secondary GO classification.

Transstadial transmission, exhibited by tick-borne pathogens, is a major adaptation promoting long-lasting persistence, and this can increase transmission efficiency. The molecular mechanisms that TBP uses to maintain transstadial transmission are not known. We examined the differential gene expression profiles of tick gut between the AT adults and CT adults under *B. microti* infection. In total, 58 and 219 differentially expressed genes (DEGs) were identified under negative (without *B. microti* infection) and positive mode (with *B. microti* infection), respectively, between AT adults and CT adults (**Figures 5A, B**).

We found that DEGs between AT adult and CT adult under negative mode are mainly involved in primary metabolic, organic substance metabolic, small molecule metabolic and NADP catabolic process (Biological Process, BP), voltage-gated channel activity, and ligase activity (Molecular Function, MF) (**Figure 5C**). *Babesia* enters ticks *via* multiple mechanisms and alters the tick gut microenvironment and the tick gut transcriptome. The DEGs between AT adults and CT adults under positive mode were mainly involved in chitin metabolic process (BP), chitin binding (MF), and the extracellular region (CC) (**Figure 5D** and **Supplement Table S4**). Chitin is a necessary component that helps maintain the barrier function of the PM in the tick gut (Kariu et al., 2013). According to the results of transcriptome and PAS staining, it is possible that this transcriptional response is microbiota dysbiosis induced by either direct or indirect action from specific bacteria. These actions thin or disrupt the PM, and pathogen infection could magnify this effect.

## Discussion

In mosquitoes, malaria parasite infection increases if the gut microbiota colonization is hindered by feeding on an antibiotic-treated sugar solution (Dong et al., 2009; Meister et al., 2009). In contrast to mosquitoes, ticks have a long-term and large-volume intake of blood. The tick microbiome can be altered by host blood meal (Swei and Kwan, 2017). Large amounts of antimicrobial agents and antibiotics have been used on animal farms to prevent and treat animal diseases, and antibiotics are widely used to treat tick bites and TBDs, such as Lyme disease, in humans. Our data showed that antibiotic used on a mouse host could alter the microbiome of adult ticks (**Figures 1**, **2**). These results suggest that antibiotics should be used reasonably to avoid the emergence and spread of antibiotic-resistant bacteria (ARB) *via* arthropods, such as ticks. If possible, antibiotics that do not promote or prevent TBDs transmission should be prioritized. We are currently studying the relationship between ARBs and the blood meal from an antibiotic treated host.

TBDs are increasing, and emerging TBPs are commonly reported. A previous study reported that the blood meal source can influence pathogen transmission (Swei and Kwan, 2017). Only the feeding stage has been examined, not the newly molted stage (Gall et al., 2016; Narasimhan et al., 2017; Swei and Kwan, 2017). Pathogens inhabiting molted unfed adult ticks mainly originate from engorged nymph ticks (similar to vertical transmission in mammals) and are transmitted to a new host during blood feeding. In this study, we investigated the microbiome and transstadial transmission of TBP in newly molted adult ticks. Our findings show that the disruption of the tick microbiome enhances tick-borne *B. microti* transstadial transmission from nymphs to adult ticks (**Figure 3**). Adult ticks are readily identified, but nymphs are easily overlooked. Our findings suggest that relatively more effort should be directed to nymph tick stage control to reduce TBD transmission.

Bacterial diversity is increased, but bacterial abundance is decreased in AT adults compared to the CT adults (**Figures 2A, C**). *Coxiella* abundance was significantly decreased in AT adults compared to CT adults. The genus *Coxiella* in ticks includes both symbiotic and pathogenic bacteria (Nardi et al., 2021), and non-pathogenic symbionts may be important in the transmission of TBPs (Bonnet et al., 2017). Li et al. (2018) suggested that *Coxiella* was likely an important defensive endosymbiont, and TBPs prevalence was decreased, with this being dependent on reduced *Coxiella* endosymbiont in ticks. We showed that the *Coxiella* abundance was decreased and *B. microti* infection increased in AT adults compared to the CT adults, which is consistent with the results of Li et al. However, the correlation between the dysbiosis of *Coxiella* in ticks and the TBPs transmission needs further study and may lead to a potential biological strategy for TBPs control.

The tick PM is considered to be a barrier to pathogen infection, although many TBPs have evolved to overcome the barrier. We found a thinner PM layer in AT adults compared to CT adults by using PAS staining (**Figure 4**). The PM is a key regulator of mosquito gut homeostasis and prevents systemic infection of malaria vector mosquitoes (Rodgers et al., 2017). In this study, the tick gut microbiota dysbiosis influenced the thickness of the PM, and the integrity of the PM was seriously disrupted after *B. microti* infection in the AT adults (**Figure 4**). The integrity of the *I. scapularis* PM affected *Borrelia burgdorferi* colonization (Narasimhan et al., 2014). We found an increase of *B. microti* infection in AT adults accompanied with a disrupted PM structure. We also compared the PM structure between the AT adults (without infection) and AT adults (with infection) and found that only the thickness but not the integrity of PM was changed in AT adults (without infection), whereas the integrity of PM was disrupted after *B. microti* infection. These results indicated that antibiotic-induced microbiome dysbiosis may affect *B. microti* infection by altering the PM structure. Furthermore, we found differential expression of PM-associated genes between AT adults and CT adults using RNA sequencing (**Figure 5**). According to these, we speculate the antibiotic disrupt the microbial homeostasis in tick midguts, that influence the PM structure in a way that regulates the expression of genes associated with PM formation, thereby resulting in a greater permissiveness to *B. microti*. The presence of gut bacteria in mosquitoes is necessary for synthesis of complete type I PM, and microbiota-induced PM prevents systemic infection of bacteria (Rodgers et al., 2017). Oral bacterial infection induced the formation of adult *Drosophila* PM, whose presence protects against intestinal bacterial infection (Kuraishi et al., 2011). These results suggest that the PM may be an important structure that is regulated by tick dysbiosis to affect *B. microti* transmission. A limitation here is that the direct association of *B. microti* and PM is not showed due to the difficulty of isolation of PM and specific bacterial. In the tick *I. scapularis*, the presence of the gut microbiota has also been shown to be necessary for production of PM proper thickness, with this being dependent on STAT-signalling (Narasimhan et al., 2014). In the mosquito, the disruption of the PM induced a systemic immune response, which is associated with the translocation of bacteria of *Enterobacteriaceae* into the body cavity (Rodgers et al., 2017). According to these reports and our present data, we speculate it is possible that tick innate immunity maybe altered due to the change of certain bacteria, and this alteration may infulence the PM structure. However, given limitations of some experimens (including the isolation and identification of specific strains, the blood feeding of ticks *in vitro*), further investigation is needed to examine how tick immunity induced by the specific bacterial modulate PM using other substitute method.

## Conclusions

Antibiotics usage in vertebrate hosts can lead to microbiota dysbiosis in ticks using these hosts for a blood meal. This dysbiosis can promote nymph-to-adult transstadial transmission of *B. microti* in *H. longicornis*. Our results indicated that PM maybe a pivotal structure which is regulated by tick dysbiosis to affect *B. microti* transstadial transmission. More studies are needed to observe the specific microbial strains in microbiota dysbiosis that influence nymph-to-adult transmission of *B. microti* and to explore the potentially safe and effective biological control strategies.

## Data Availability Statement

The data presented in the study are deposited in the Sequence Read Archive (SRA) repository, accession number (SRP322057 and SRP323180).

## Ethics Statement

The animal study was reviewed and approved by the Institutional Animal Care and Use Committee of Shanghai Veterinary Research Institute.

## Author Contributions

NW designed the projects, collected and analyzed the data, and drafted the manuscript. YZ and JC helped in the mouse housed experiments. HZ participated in the collection of tick samples. JZ participated in the design of this study and revision the manuscript. All authors contributed to the article and approved the submitted version.

## Funding

This work was sponsored by Shanghai Sailing Program (19YF1458600) and the China Postdoctoral Science Foundation (2018M641567).

## Conflict of Interest

The authors declare that the research was conducted in the absence of any commercial or financial relationships that could be construed as a potential conflict of interest.

## Publisher’s Note

All claims expressed in this article are solely those of the authors and do not necessarily represent those of their affiliated organizations, or those of the publisher, the editors and the reviewers. Any product that may be evaluated in this article, or claim that may be made by its manufacturer, is not guaranteed or endorsed by the publisher.
