# Corrigendum: The Tick Microbiota Dysbiosis Promote Tick-Borne Pathogen Transstadial Transmission in a *Babesia microti* Infected Mouse Model

**DOI:** 10.3389/fcimb.2021.765387

**Published:** 2021-09-27

**Authors:** Nana Wei, Jie Cao, Houshuang Zhang, Yongzhi Zhou, Jinlin Zhou

**Affiliations:** Key Laboratory of Animal Parasitology of Ministry of Agriculture, Shanghai Veterinary Research Institute, Chinese Academy of Agricultural Sciences, Shanghai, China

**Keywords:** *Haemaphysalis longicornis*, microbiota dysbiosis, antibiotic usage, *Babesia microti*, peritrophic matrix

In the original article, there was a mistake in the legend for **Figure 2** as published. OTUs should be changed to ASVs. The correct legend appears below.

**Figure 2:**
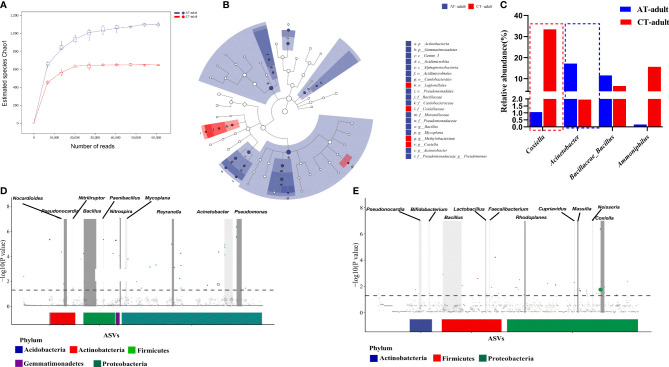
Antibiotics used on mice resulted in tick microbiota dysbiosis. **(A)** Rarefaction curve of the estimated number of genera using the Chao1 method. **(B)** Cladogram for taxonomic representation of significant differences between two groups. **(C)** Percentages of the most abundant genera in AT adults and CT adults. **(D, E)** Manhattan plots showing the abundance enrichment in the AT adults **(D)** and CT adults **(E)**. The dashed line represents the P = 0.05 threshold of significance.The color represents the different taxonomic affiliation of the ASVs (phylum level), and the dot size corresponds to their relative abundance in the respective samples. Gray boxes denote the different taxonomic groups (genus level).

“The color represents the different taxonomic affiliation of the ASVs (phylum level), and the dot size corresponds to their relative abundance in the respective samples.”

The authors apologize for this error and state that this does not change the scientific conclusions of the article in any way. The original article has been updated.

In the original article, there was a mistake in **Figure 2** as published. OUTs should be changed to ASVs. The corrected **Figure 2** appears below.

The authors apologize for this error and state that this does not change the scientific conclusions of the article in any way. The original article has been updated.

In the original article, there was a mistake in **Supplementary Material** as published. **Supplement Table S3** in the **Data sheet 1** showed wrong (showed repeat with **Table S1** due to a typographical error). The corrected **Supplement Table S3** appears below.

**Table S3:** Parasitemia density of *B. microti* in mice at different time points.

Mice-model group	Mouse ID	Day 0 (%)	Day 3 (%)
Antibiotic treated	1	15.67	50.72
	2	19.34	49.76
	3	21.6	49.14
Control	1	12.6	51.54
	2	18.02	51.7
	3	16.78	49.76

The authors apologize for this error and state that this does not change the scientific conclusions of the article in any way. The original article has been updated.

## Publisher’s Note

All claims expressed in this article are solely those of the authors and do not necessarily represent those of their affiliated organizations, or those of the publisher, the editors and the reviewers. Any product that may be evaluated in this article, or claim that may be made by its manufacturer, is not guaranteed or endorsed by the publisher.

